# Molecular Docking and Dynamics Simulation of Several Flavonoids Predict Cyanidin as an Effective Drug Candidate against SARS-CoV-2 Spike Protein

**DOI:** 10.1155/2022/3742318

**Published:** 2022-11-09

**Authors:** Asmita Shrestha, Rishab Marahatha, Saroj Basnet, Bishnu P. Regmi, Saurav Katuwal, Salik Ram Dahal, Khaga Raj Sharma, Achyut Adhikari, Ram Chandra Basnyat, Niranjan Parajuli

**Affiliations:** ^1^Central Department of Chemistry, Tribhuvan University, Kirtipur, Kathmandu 44618, Nepal; ^2^Department of Chemistry, Oklahoma State University, Stillwater, OK 74078, USA; ^3^Center for Drug Design and Molecular Simulation Division, Cancer Care and Research Center, Kathmandu, Nepal; ^4^Department of Chemistry, Florida Agricultural and Mechanical University, Tallahassee, FL 32307, USA

## Abstract

The *in silico* method has provided a versatile process of developing lead compounds from a large database in a short duration. Therefore, it is imperative to look for vaccinations and medications that can stop the havoc caused by SARS-CoV-2. The spike protein of SARS-CoV-2 is required for the viral entry into the host cells, hence inhibiting the virus from fusing and infecting the host. This study determined the binding interactions of 36 flavonoids along with two FDA-approved drugs against the spike protein receptor-binding domain of SARS-CoV-2 through molecular docking and molecular dynamics (MD) simulations. In addition, the molecular mechanics generalized Born surface area (MM/GBSA) approach was used to calculate the binding-free energy (BFE). Flavonoids were selected based on their *in vitro* assays on SARS-CoV and SARS-CoV-2. Our pharmacokinetics study revealed that cyanidin showed good drug-likeness, fulfilled Lipinski's rule of five, and conferred favorable toxicity parameters. Furthermore, MD simulations showed that cyanidin interacts with spike protein and alters the conformation and binding-free energy suited. Finally, an *in vitro* assay indicated that about 50% reduction in the binding of hACE2 with S1-RBD in the presence of cyanidin-containing red grapes crude extract was achieved at approximately 1.25 mg/mL. Hence, cyanidin may be a promising adjuvant medication for the SARS-CoV-2 spike protein based on *in silico* and *in vitro* research.

## 1. Background

As the COVID-19 pandemic is at the end of its third year, public health officials must assess where we are and how we may break the SARS-CoV-2 devastating grip in the world. The rapid discovery of many safe and effective COVID-19 vaccines has been one of the pandemic's biggest scientific achievements. However, vaccines alone will not be enough to stop the pandemic due to more transmissible new variants, and vaccines are designed to guard against severe suffering and death [1]. New variants due to mutation in SARS-CoV-2 are a subject of concern because the emerging mutants have a potential for enhanced infectivity, competitive fitness, and transmission [2]. There occurs an accumulation of mutations, which drives viral evolution and genome variability, and this enables the virus to escape host immunity and develop drug resistance [3]. Important mutations have appeared in the SARS-CoV-2 spike protein that interacts with the host immune system [4]. The reported variant, omicron (B.1.1.529) [5], has 30 mutations in the spike protein along with K417N, which was earlier recognized to reduce the effectiveness of a cocktail of therapeutic monoclonal antibodies [6].

The SARS-CoV-2 spike protein is a heavily glycosylated homotrimeric transmembrane protein; the S1 subunit contains an RBD that facilitates viral attachment to the host receptor angiotensin-converting enzyme-2 (ACE2), and the S2 subunit mediates viral entry by mediating host-viral membrane fusion [7]. The biomechanical strength of the ACE2-spike protein manages viral adherence and access to host cells [8]. Hence, the spike protein of SARS-CoV-2, which plays a crucial role in viral attachment and fusion, could be a potential therapeutic target.

Several strategies have been used to develop antiviral drugs for SARS-CoV-2; to date, some drugs are effective against this virus, but only some neutralizing antibodies and remdesivir have been approved by the US FDA [9]. The drug development against SARS-CoV-2 is mainly focused on interrupting the virus's life cycle by blocking its interaction with the host [10, 11]. An increasing number of recent studies have used computational methods for identifying new drug targets or drug repurposing candidates. Nowadays, various natural compounds have been repurposed/tested using computer-aided drug discovery programs [12, 13]. *In silico* approaches are cost-effective and actionable approaches that can be used to screen several compounds, enabling the discovery of drug combinations and novel drugs.

Flavonoids are natural phytochemicals with antiviral properties which have been discovered to inhibit different targets of SARS-CoV [14] such as interfering with spike protein and blocking enzymatic activities of viral proteases, 3-chymotrypsin-like proteases (3CL^pro^), and papain-like proteases (PL^pro^). Previous research suggests that methylated flavonoids, such as retusin, could be used as an antiviral or adjuvant medication in the treatment of COVID-19 [15]. Flavonoids are promising plant-derived chemicals for treating SARS-CoV-2 infection, either through direct antiviral effects or by controlling the host immunological response to viral infection [16]. Previously, several research studies have investigated the use of flavonoids against SARS-CoV-2 [16]. An *in silico* technique was used to examine and compare several flavonoids known to have anti-inflammatory and antiviral characteristics in an attempt to suppress the spike glycoprotein of SARS-CoV-2, revealing naringin as a potential therapeutic candidate for COVID-19 [17]. Based on molecular docking and ADMET analysis, flavonoids, such as cyanidin-3-(p-coumaroyl)-rutinoside-5-glucoside, delphinidin-3-O-beta-D-glucoside 5-O-(6-coumaroyl-beta-D-glucoside), albireo delphine, apigenin 7-(6”-malonylglucoside), and (-) maackiain-3-O-glucosyl-6”-O-malonate were demonstrated as potent inhibitors against the spike protein, 3CLpro, and RdRP of SARS-CoV-2 [18]. Furthermore, molecular docking and simulation studies on flavonoid-protein complexes, including luteolin-spike protein and mundulinol-spike protein, have been found to demonstrate strong interactions compared to recently used FDA-approved drugs such as favipiravir, hydroxychloroquine, and lopinavir [19]. Flavonoids, such as dorsilurin E, euchrenone a11, and sanggenol O, were found to inhibit SARS-CoV-2 main protease (M^pro^) [20]. Similarly, cyanidin and quercetin were found to inhibit the RNA polymerase function and block interaction sites of the spike protein [21, 22]. The 3CL-protease inhibition assay, cytotoxicity study, total flavonoid assay, and molecular docking study were all used to examine a mixture of 11 flavonol glycosides prepared from *S. persica*. It was discovered that these compounds inhibited M^pro^, spike protein fusing onto its allosteric site [23]. *In vitro* assay of the extract prepared from muscadine grapes showed inhibitory activity against the M^pro^ of SARS-CoV-2 [24]. The foundation for future research has been laid by earlier studies of flavonoids' effects on SARS-CoV and SARS-CoV-2 inhibition.

Flavonoids, an important class of natural products, can affect CoVs at the initial phase of the entrance, replication, and virion release from the host cells [25]. A stable complex between the spike protein and flavonoids is expected to form due to ortho di-OH hydroxyl groups in the B ring of flavonols [26]. Many flavonoids have been found to block the life cycle of multiple CoV targets (spike protein, proteases, TMPRSS2, etc.) through various mechanisms [27, 28]. Flavonoids, therefore, can act as prophylactic, therapeutic, or indirect inhibitors [29]. Flavonoids in this research were chosen based on previously reported *in vitro* assay data (Table S1) to assemble cyanidin and other flavonoids as promising drug candidates against SARS-CoV-2. Furthermore, *in vitro* assay on the crude extract of red grapes has been carried out for the validation of computational predictions.

## 2. Materials and Methods

### 2.1. Preparation and Molecular Modelling of the Target Protein

The crystal structure of the SARS-CoV-2 RBD of spike glycoprotein (PDB ID: 7NX8) with a resolution of 1.95 Å was retrieved from the Protein Data Bank (PDB) (Table S2). The obtained protein was mutated by replacing threonine (T) with asparagine (N) at position 417 through a sequence editor utilizing the protein builder in Molecular Operating Environment (MOE) software (version 2020.0901). The structure was optimized using the MOE protein preparation module by removing water molecules, adding hydrogen atoms, and assigning atomic charges to all protein atoms. Moreover, energy minimization was done using different force field parameters of the MOE preparation module.

### 2.2. Preparation of Ligands

**Figure 1:**
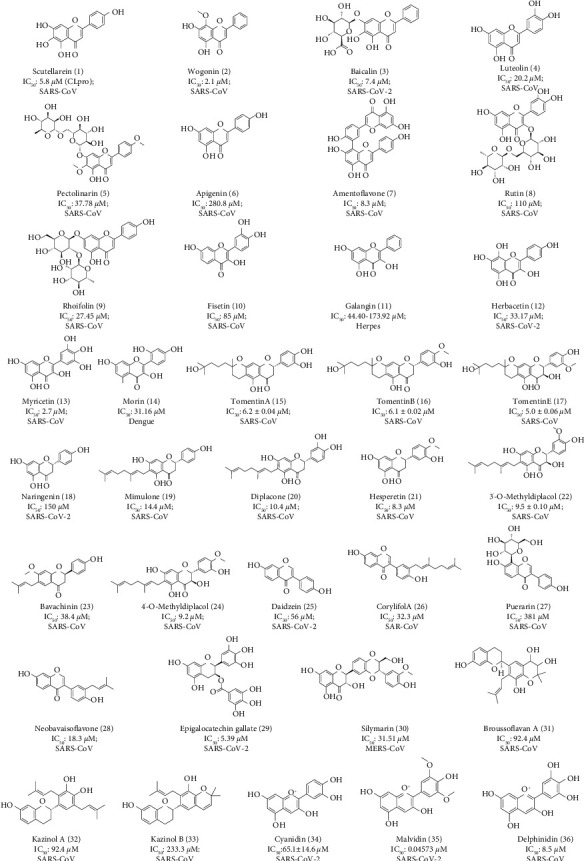
Chemical structures of selected antiviral flavonoids with their IC 50 values against different viruses according to *in vitro* approach done in previous studies.

After a comprehensive literature review, the flavonoids were selected based on their antiviral properties against SARS-CoV and SARS-CoV-2, and the structures were accessed from the PubChem database [30] and Chem Spider [31]. Finally, the chosen flavonoids were processed into mol2 file format using open babel, and the energy minimization for molecular docking was done using the MOE software [32]. The structures of the selected 36 natural antiviral flavonoids and their IC_50_ values against several viruses, including SARS-CoV, SARS-CoV-2, and dengue, are shown in Figure 1.

### 2.3. Active Site Prediction

**Figure 2:**
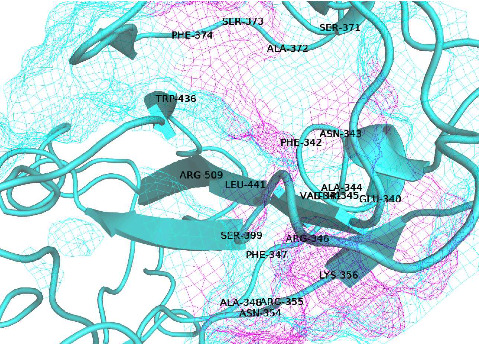
The binding pocket of spike protein along with binding residues.

MOE Site Finder, which is based on the concept of alpha spheres, was used to determine the amino acid residues involved in the active pocket of the spike protein, where the relative positions and accessibility of the receptor atoms were considered [33]. Furthermore, using the site finder, the number and name of the residues of the active site were predicted.  Table 1 shows the detected cavities in the spike protein region.  Figure 2 shows the binding cavity of the spike protein, showing the binding site along with binding residues.

### 2.4. Drug-likeness and ADMET Studies

Identification of drug-like characteristics of the selected flavonoids was performed using Lipinski's rule of 5 [34] and the SwissADME web tool [35]. ADMET (Adsorption, Desorption, Metabolism, Excretion, and Toxicity) studies and toxicity prediction were assessed through the pkCSM web server [36] and ProTox-II [37], respectively.

### 2.5. Molecular Docking and Validation

MOE and GOLD (genetic optimization for ligand docking) (version 4.0.1) software packages [38] were used to predict the protein-ligand interactions. MOE docking suite uses the virtual screening protocol of MOE in combination with the triangle matcher docking algorithm and London dG scoring function, and GOLD is based on a genetic algorithm. The docking analysis was performed on a Microsoft Windows workstation (Intel Core i5-9400 CPU processor and system memory 4 GB RAM). A molecular docking study was conducted on selected flavonoids with SARS-CoV-2 spike protein that carries the K417N mutation. The docking results were validated by extracting the commercial drug and top-scored metabolites from their original binding site and redocking them into the same position using the GOLD default docking protocol [39]. MOE provides S-score as its scoring function. The best pose obtained from MOE is further processed into GOLD software, and finally, the GOLD score is obtained. To validate the docking results, the lowest energy pose obtained on redocking and the previous docking positions of the metabolites were superimposed, and its root means square deviation (RMSD) was calculated.

### 2.6. Molecular Dynamics Simulation

The GROMACS tool was used to produce the protein topology file and parameterize the natural flavonoids' ligand topology. MD simulation for 100 ns was performed in GROMACS 5.1.1 using GROMOS 43a1 force field for the protein-ligand system and HSA-ligand to clarify the facts behind the efficiency of this ligand in protein inhibition [40]. The PRODRG server was used to obtain a required file of ligand [41]. During the initial step of the simulation, a cubic box was generated around the protein-ligand complex and solvated with Simple Point Charge (SPC) at the range of 1.0 nm between the wall of components of the protein complex [42]. Sodium ion (Na^+^) and chloride ion (Cl^−^) with an ionic strength of 0.1 M were added to neutralize the solvated system. The system's energy was minimized through 50,000 steps of the steepest descent approach, followed by an equilibrium process using Berendsen thermostat (NVT) ensembles. The integral time phase was two femtoseconds (fs), and the neighbor list was updated for every twentieth step with a cut-off range of 12 Å using the grid option. Periodic boundary conditions (PBCs) were used with a constant number of particles in the system, constant pressure, and constant temperature (NPT) simulation criteria. Equilibration of the system at 1 bar pressure for 1 ns was connected using a Parrinello−Rahman barostat in this simulation [42, 43]. Using trajectories obtained from MD simulations, root mean square Deviation (RMSD), root mean square fluctuation (RMSF), and the radius of gyration (Rg) were analyzed. The average structure of the complex was estimated within the last 10 ns trajectory of MD simulations before the RMSF computation, and then each residue around the ligand was aligned to the average structure. During the simulation, the stability of the C-alpha atoms in amino acids was considered. The stability of the MD trajectories was investigated using the backbone RMSD values of atoms about the spike protein complex, and the time evolution plot of Rg was computed to estimate the conformational stability of the protein-ligand. The RMSF of all the amino acids around the ligand at 1 nm was determined using the VMD software to investigate the conformational flexibility of the leading active site during the simulation procedure. The MD runs were executed on an AMD processor (32 cores/64 threads) with 128 GB RAM. The visual analysis of RMSD, RMSF, and Rg was done using VMD.

### 2.7. Calculation of Binding Free Energy (BFE)

The prime MM-GBSA [44] of the Schrodinger suite with the OPLS force field is a popular method to calculate the BFE of binding ligands to proteins. This approach is based on docking a ligand and a protein and calculating binding energy using the following equation [45]:

(1) $$\begin{array}{c} \Delta G_{\text{bind}}=\Delta E+\Delta G_{\text{Solv}}+\Delta G_{SA}\text{,} \end{array}$$

where ∆G_bind_ is the BFE of the protein-ligand system and ∆*E* is the difference in the minimized energies between the protein-ligand complex and the sum of the energies of the free protein and ligand. ∆G_Solv_ is the difference in the GBSA solvation energy of the protein-ligand complex and the sum of the solvation energies for the free protein and free ligand. ∆G_SA_ is the difference in surface area energies for the complex and the sum of the surface area energies for the free protein and free ligand. To prioritize the lead inhibitors, the MM-GBSA technique was applied as a rescoring function. To optimize the molecules and choose the best compounds, BFE obtained from prime MM/GBSA calculations was considered, along with the docking scores.

### 2.8. *In Vitro* Spike S1-RBD and ACE2 Inhibitory Activity of SARS-CoV-2 by Enzyme-Linked Immune Sorbent Assay (ELISA)

The crude methanolic extract of red grape (*Vitis vinifera*) was prepared with 32.5% yield by the maceration method described elsewhere [46]. A 96-well plate coated with recombinant 2019-nCoV S1-RBD [47] (catalog no. PR-ncov-2-PL, NOVATEINBIO) was added with increasing concentrations of crude extract to determine whether it inhibits the interaction between hACE2 and SARS-CoV-2 S1-RBD. For each concentration, the assay was performed in triplicate, and % inhibition was expressed as a mean ± standard error of the mean of triplicate. After incubation for 2 hours at 37°C and then washing for 3×PBS (pH 7.2), plates were blocked with 1% BSA and 0.05% Tween-20 in PBS. After washing 3 times, 100 *μ*L hACE2 receptor protein (catalog no.: PR-nCoV-4) (0.1–0.2 *μ*g/mL) was added and incubated for 1 hour in the binding buffer (0.1% BSA in PBS, pH 7.2). Then, 100 *μ*L goat anti-human IgG-Fc, HRP conjugated 1 : 500 in binding buffer was added after washing plates three times. Again, after three washes by washing buffer, 3,3′,5,5′-tetramethylbenzidine (TMB) was added for the signal and the reaction was stopped by adding an acidic solution. The plates were read at 450 nm for absorbance, and % inhibition was calculated using GraphPad.

## 3. Results

### 3.1. Pharmacokinetic Profiles of Flavonoids

All 36 flavonoids were chosen to have a high absorption rate. In addition, their skin permeability, the volume of distribution at steady-state (VDss), CNS permeability, and blood-brain barrier (BBB) permeability were investigated because they play a crucial role in determining drug distribution. Among different cytochromes P450 (CYPs) enzymes, the main focus of our study was human cytochrome P450 3A4 (CYP3A4), which was found to be inhibited by flavonoids 1, 2, 4, 6, 10, 11, 16, 19, 23, 26, 28, 32, 33, 34, and 35 indicating that they may be metabolized in the liver shown in Table S3. The toxicity of the selected flavonoids was also predicted using ProTox-II, which computes median lethal dose (LD_50_) values and toxicity classes.  Table S4 displays LD_50_ values and toxicity classes of 37 flavonoids predicted by using ProTox-II. In regard of acute oral toxicity, flavonoids numbering 1–9, 11, 12, 14, 25, 26, 28, 31, 32, 34, 35, and 36 are found to fall in toxicity class V (2000 < LD_50_ ≤ 5000), while 17 in VI (LD_50_ > 5000). The flavonoids 10 and 13 fall in toxicity class III (50 < LD_50_ ≤ 300), and the remaining belong to toxicity class IV (300 < LD_50_ ≤ 2000). In this investigation, most of the flavonoids passed Lipinski's criteria, indicating that they are safe to use as drugs.  Table S5 shows the ADME molecular descriptors of the selected flavonoids designed to inhibit SARS-CoV-2 by the SwissADME server.  Table 2 shows the five key physiochemical parameters of the selected 6 flavonoids which are used to test Lipinski's rule of 5 to evaluate drug-likeness, and Table 3 shows the ADMET profiles of the selected 6 flavonoids.

### 3.2. Molecular Docking Analysis

**Figure 3:**
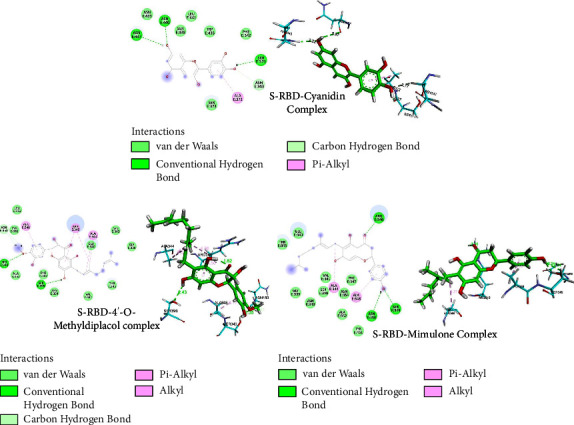
2D and 3D structures of cyanidin, 4′-O-methyldiplacol, and mimulone complexed with SARS-CoV-2 spike protein.

As indicated earlier, we used the K417N mutant of SARS-CoV-2 spike protein in molecular docking analysis as the mutation at the 417 position by asparagine (N) reduces the therapeutic efficacy of a combination of monoclonal antibodies [6]. The IC_50_ values, GOLD fitness score, bond length, and spike protein interacting residues for the top-scored flavonoids along with FDA-approved drugs, lopinavir, and remdesivir, are presented in Table 4. A prior *in vitro* investigation on SARS-CoV-2 identified cyanidin as a promising treatment candidate, and the current study shows that it has an appropriate GOLD fitness score of 51.91, indicating its potency as the spike protein inhibitor. Furthermore, 4′-*O*-methyldiplacol, mimulone, neobavaisoflavone, malvidin, and tomentin E also interact appropriately with the binding site of the spike protein with GOLD fitness scores of 63.83, 61.60, 53.90, 52.01, and 50.91, respectively. As shown in Figure 3, cyanidin interacts with the spike protein through Asn 343, Ser 371, Asn 437, Asn 440, and Ser 372 via hydrogen bonds and hydrophobic interactions. Similarly, 4′-*O*-methyldiplacol, mimulone, malvidin, tomentin E, and neobavaisoflavone interact with the protein through different residues shown in Table 4. The GOLD fitness score, interacting residues of the protein, and interaction distances of all remaining flavonoids studied are displayed in Table S6. Similarly, Figure S1 depicts the 2D and 3D structures of the remaining 4 potential docked protein-ligand complexes.

### 3.3. Molecular Dynamics Simulation Analysis

**Figure 4:**
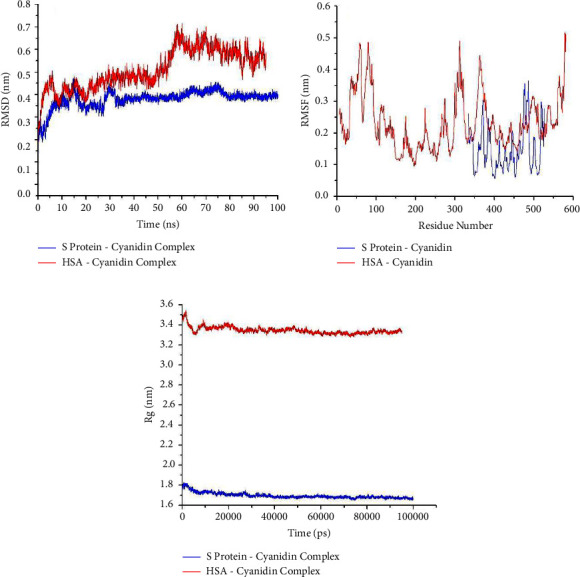
(a) Plot of RMSD, (b) RMSF, and (c) Rg during 100 ns MD simulation of spike protein-cyanidin complex and HSA-cyanidin complex.

MD simulations were run on the docked complex to learn more about the ligand-protein interactions. The RMSD values of the spike-cyanidin complex and human serum albumin (HSA)-cyanidin complex are shown in Figure 4(a)). The average RMSD fluctuation for the protein and ligand in the spike-cyanidin complex is 0.39 nm, with equilibrium after 80 ns. The RMSD of cyanidin was found to be stable first from 36 to 60 ns and later from 80 ns to 100 ns. Similarly, large deviations in RMSD were observed for the HSA-cyanidin complex, indicating an unstable nature of the complex thus formed. These observations support that cyanidin binds with the spike protein rather than HSA. The RMSF was measured to further compute the residual flexibility over 100 ns shown in Figure 4(b). The RMSF is less than 0.36 nm for each residue surrounding the ligand in the spike protein complex, while for HSA, it is ∼0.5 nm. The *R*g trajectory of spike-cyanidin reaches equilibrium at ~ 35 ns and is steady during 35–100 ns, indicating that the ligand is effectively fitted at the active site of spike protein [Figure 4(c)]. In contrast, the *R*g trajectory of HSA-cyanidin optimizes equilibrium during ∼60–80 ns. According to *R*g plots, the structural compactness of spike protein-ligand remains stable with an average *R*g value of 1.6 nm; in contrast, the HSA-cyanidin complex indicates instability with an average *R*g value of 3.5 nm. Conclusively, the lower the *R*g value, the higher the compactness of protein-ligand, resulting in a stronger interaction between them [53, 54].

### 3.4. Binding Free Energy (BFE) Analysis

To quantify the response of active residues with ligands, we performed the prime MM/GBSA method, which calculates the absolute BFE to determine the strength of the interaction of the ligand with the protein. The flavonoids cyanidin, mimulone, and 4′-*O*-methyldiplacol display significantly lower binding energy (ΔG_bind_ −25.09, −21.23, and −18.40 kcal/mol, respectively), indicating that they strongly bind with the protein. BFE calculation revealed the stable binding of cyanidin with spike, corroborating the molecular docking and conformational dynamics analyses.

### 3.5. *In Vitro* Assays

The red grapes crude extract containing cyanidin [55, 56] was *in vitro* tested using enzyme-linked immunosorbent assay in response to promising *in silico* results that revealed that cyanidin could inhibit the interaction between hACE2 and SARS-CoV-2 S1-RBD. A series of concentrations of extract was incubated with hACE2 on 96-well plates coated with S1-RBD, and the signal was generated using an HRP-linked secondary antibody. Notably, we determined that red grapes extract had the potential to inhibit the interaction of hACE2 with S1-RBD protein. The detail of the measurement of absorbance and percent inhibition for binding of human ACE2 to S1-RBD protein is shown in Table S7. About 50% reduction in the binding of hACE2 with S1-RBD in presence of red grapes crude extract was achieved at approximately 1.25 mg/mL (Figure S2). Altogether, this approach provides significant mediating evidence that cyanidin, a major flavonoid in red grapes (*Vitis vinifera*), may inhibit the binding of hACE2 and SARS-CoV-2 S1-RBD.

## 4. Discussion

Despite continued efforts from various research groups, no effective drug against COVID-19 has been developed yet. In the search for a safe and effective drug, the computational approach provides a good platform for the virtual screening of metabolites to select potential drug candidates [57]. Therefore, repurposing natural secondary metabolites could be the most effective way to discover a remedy for COVID-19 infection [58]. To predict the pharmacokinetic properties of potential drugs, ADMET is an important parameter to be considered. For effective metabolism and activity, a good drug candidate should be absorbed in a specific time frame and properly distributed throughout the system. Cytochrome P450s (CYPs) play a key role in phase I of drug biotransformation. Five CYPs (1A2, 2C9, 2C19, 2D6, and 3A4) mediate about 95% of CYP-mediated drug metabolism in the body [59]. ADMET properties of metabolites are analyzed and short-listing of metabolites is done by using the Lipinski rule of five, which determines their drug-likeliness property. According to the Lipinski rule, metabolites with a drug-like property should have a molecular weight (mol·wt.) ≤ 500, hydrogen bond acceptor (HBA) ≤ 10, and the hydrogen bond donor (HBD) should be ≤ 5 [60]. Due to the structural diversity of flavonoids, they exhibit versatile biological benefits such as anti-inflammatory, neuroprotective, and antioxidative, as well as antiviral properties [17]. Different specific flavonoids have been reported effective as antiviral agents for some viruses such as dengue, hepatitis C virus, and influenza by the modification of viral proteins, inhibition of viral entry, and inhibition of viral neuraminidase [61]. The most investigated targets for flavonoid inhibition are proteases (3CL^pro^ and PL^pro^) of SARS-CoV and MERS-CoV [62]. Flavonoids such as kaempferol are reported to inhibit cation-selective channels formed by the ORF 3a channel of SARS-CoV. As a result, it ultimately inhibits the virus release and stands out as the root of the evolution of new medicinal antiviral drugs [63].

SARS-CoV-2 S-RBD interacts with host receptor ACE-2 via RBD, laying the groundwork for viral entrance in receptor cells [64]. The emerging variants of SARS-CoV-2 are connected to the mutation of its spike protein, which is important for the entrance of the virus into the host cells [65]. So, our study considered the spike protein as the target protein for molecular docking of flavonoids having an antiviral activity to analyze the GOLD score and binding interactions of metabolites. The GOLD score of 4′-*O*-methyldiplacol, mimulone, and cyanidin was found to be 63.83, 61.60, and 51.91, respectively. The greater the GOLD score, the greater its protein-ligand interactions [66]. The strong interaction of selected ligands to the protein can result in the conformational change of the viral protein and hence, inhibit its replication or growth. However, we considered cyanidin as a promising candidate among the previous three, as it follows Lipinski's rule of 5 and has been revealed to inhibit the SARS-CoV-2 protein in its derivative form with an IC_50_ value of 65.1 ± 14.6 *μ*M [48]. In addition, cyanidin shows minimum binding energy with the target protein. It is also reported that cyanidin has around 92% inhibition at 150 *μ*M for M^pro^ enzymatic assay [67]. In our study, cyanidin interacted with the spike protein forming four hydrogen bonds (with residues Asn 343, Ser 371, Asn 437, and Asn 440) and one pi-alkyl bond (with residue Ala 372); 4′-*O*-methyldiplacol forms three hydrogen bonds (with residues Ser 399, Ser 349, and Asn 450) and three pi-alkyl bond (with residues Ala 348, Arg 346, and Ala 344); mimulone forms three hydrogen bond (with residues Arg 346, Ser 349, and Asn 450) and two pi-alkyl bond (with residues Ala 344 and Ala 348) which may result in its conformational change. The docking algorithms used in this study provide several binding poses. The selection of the best docking pose is carried out based on the number of H-bond present, hydrophobic interactions [68], interactions with the active sites, and binding energy values. Besides, molecular flexibility, binding scoring, and computing time can be a hurdle in any molecular docking approach [69]. Hence, further MD simulations and binding energy calculations were performed to alleviate the limitations of docking results.

To assess MD trajectories, the RMSD is a crucial calculation. Using the RMSD of a protein-ligand complex system, the average distance generated by the dislocation of a given atom over time is calculated [70]. The RMSD of cyanidin was determined to be stable first from 36 to 60 ns and later from 80 ns to 100 ns. The RMSF is used to calculate the flexibility among amino acid residues. It is important for monitoring local protein alteration since it allows the calculation of the average change detected over a large number of atoms to determine the displacement of a given atom relative to the reference structure [71]. The binding pocket was found relatively stable during the MD simulations revealed through obtained RMSF for each residue surrounding the ligand in the protein complex. *R*g reflects the ligand-protein complex compactness, with a smaller radius of gyration indicating a more compact structure [72]. From the calculation of the *R*g, cyanidin effectively binds with the spike protein and found that the complex system was suitably stable ranging from 1.675 nm to 1.70 nm. In addition, the MD simulation analysis of the spike protein-cyanidin complex was compared with the HSA-cyanidin complex which reveals the stability of the cyanidin complexed with the target protein. It concludes that the cyanidin comes to bind with the spike protein rather than HSA.

## 5. Conclusion

We have used a computational chemistry protocol to identify the most promising flavonoids with *in vitro* IC_50_ values that inhibit SARS-CoV and SARS-CoV-2 activity. This protocol includes ADMET analysis, molecular docking, molecular dynamics simulations, and BFE calculations to predict whether these flavonoids are suitable for anti-COVID-19 therapy. This research further enhances that cyanidin interacted with SARS-CoV-2 spike protein, exhibiting the best binding poses and forming stable protein-ligand complexes and lower binding-free energy. Moreover, 4′-*O*-methyldiplacol and mimulone also showed promising data, based on molecular docking analysis, but as it falls under toxicity class IV, this study repelled to take them as potent agents. Additionally, *in vitro* study on the crude extract of red grapes ensured the candidacy of cyanidin as an antiviral drug against SARS-CoV-2. Based on the furnished shreds of evidence, further clinical research on cyanidin and its large-scale clinical trials and mouse model tests are suggested.

## Figures and Tables

**Table 1:** Active site residues of spike protein using the Site Finder module.

The binding site of spike protein	Size of polypeptides	Amino acid residues
1	32	Glu 340, Val 341, Ala 344, Thr 345, Arg 346, Phe 347, Ala 348, Asn 354, Arg355, Lys 356, Ser 399

2	20	Phe 342, Asn 343, Ala 344, Thr 345, Ser 371, Ala 372, Ser 373, Phe 374, Trp 436, Leu 441, Arg 509

3	16	Leu 335, Cys 336, Pro 337, Phe 338, Gly 339, Asn 343, Val 362, Ala 363, Asp 364, Tyr 365, Leu 368, Ser 371

**Table 2:** Physiochemical parameters of the selected 6 flavonoids predicted by using SwissADME.

Parameters	Mass (<500)	Hydrogen bond donor (<5)	Hydrogen bond acceptor (<10)	Log*P* (<5)	Molar refractivity (40–130)
4′-*O*-Methyl diplacol	454.51	4	7	4.32	126.51
Mimulone	408.49	3	5	4.80	118.85
Neobavaisoflavone	322.35	2	4	3.74	95.69
Malvidin	331.30	4	7	0.71	87.13
Cyanidin	287.24	5	6	0.56	76.17
Tomentin E	472.53	4	8	3.24	126.24

**Table 3:** ADMET profiles of the selected 6 flavonoids predicted by using pkCSM and ProTox-II.

Parameters	Blood-brain barrier (BBB)	Human intestinal absorption (HIA)	CYP3A4 inhibitor	AMES toxicity	Hepato toxicity	LD_50_	Toxicity class
4′-*O*-Methyl diplacol	−1.311	76.952	No	No	No	2000	IV
Mimulone	−1.056	90.287	Yes	No	No	2000	IV
Neobavaisoflavone	−0.09	94.31	Yes	No	No	2500	V
Malvidin	−1.560	71.558	Yes	No	No	5000	V
Cyanidin	−1.357	80.203	Yes	Yes	No	5000	V
Tomentin E	−1.396	77.239	No	Yes	No	10000	VI

**Table 4:** Binding free energies, GOLD fitness score, interacting residues of spike protein with their interaction distance, and IC_50_ values of potent metabolites which were obtained from previous studies through *in vitro* analysis.

Compounds	Binding free energies (Kcal/mol)	GOLD fitness score	Interacting residues of the spike protein	Interaction distance (Å)	IC_50_ value (*μ*M) and citations
Cyanidin	−25.09	51.91	Asn 343	2.77	65.1 ± 14.6 *μ*M (SARS-CoV-2) [48]
			Ser 371	2.37	
			Ser 372	4.16	
			Asn 437	2.97	
			Asn 440	2.22	

Malvidin	−22.03	52.01	Arg 346	2.72	0.04573 *μ*M (SARS-CoV-2) [49]
			Phe 347	1.83	
			Ser 349	2.24	
			Asp 442	2.68	
			Lys 444	2.20	

Tomentin E	−25.02	50.91	Glu 340	2.30	5.0 *μ*M (SARS-CoV) [50]
			Lys 356	2.37	
			Ser 399	2.07	

4′-O-Methyldiplacol	−18.40	63.83	Ala 344	4.05	9.2 *μ*M (SARS-CoV) [50]
			Arg 346	4.36	
			Ala 348	3.77	
			Ser 349	2.35	
			Ser 399	2.43	
			Asn 450	2.52	

Mimulone	−21.23	61.60	Ala 344	4.27	14.4 *μ*M (SARS-CoV) [50]
			Arg 346	2.40	
			Ala 348	3.90	
			Ser 349	2.24	
			Asn 450	2.54	

Neobavaisoflavone	−19.49	53.90	Ser 469	2.72	18.3 *μ*M (SARS-CoV) [51]
			Gln 474	2.54	

Lopinavir	−22.75	59.82	Gly 339	4.23	26.63 *μ*M (SARS-CoV-2) [52]
			Val 341	4.97	
			Ala 344	5.44	
			Arg 346	2.10	
			Phe 347	2.05	
			Asn 354	2.16	
			Lys 356	5.10	

Remdesivir	−18.03	56.33	Leu 335	5.47	23.15 *μ*M (SARS-CoV-2) [52]
			Pro 337	2.44	
			Gly 339	2.44	
			Asn 343	2.21	
			Asp 364	2.15	
			Val 367	4.41	

## Data Availability

Molecular docking and MD simulation data generated in this research will be provided upon request to the corresponding author.

## Abbreviations

SARS-CoV-2: Severe acute respiratory syndrome coronavirus 2
RBD: Receptor binding domain
MD: Molecular dynamics
MM/GBSA: Molecular-mechanics generalized born surface area
BFE: Binding-free energy
COVID-19: Coronavirus disease 19
ACE-2: Angiotensin-converting enzyme 2
ADMET: Adsorption, distribution, metabolism, excretion, and toxicity
PDB: Protein Data Bank
MOE: Molecular operating environment
GOLD: Genetic Optimization for Ligand Docking
RMSD: Root mean square deviation
RMSF: Root mean square fluctuation
*R*g: Radius of gyration
HSA: Human serum albumin
ELISA: Enzyme-linked immunosorbent assay
TMB: 3,3′,5,5′-Tetramethylbenzidine.

## Supplementary Materials

Supplementary Materials Table S1: plant secondary metabolites (flavonoids) with their antiviral activity. Table S2: target protein with PDB ID, resolution, and description of the protein selected for docking with complexed inhibitor. Table S3: ADMET properties of selected flavonoids using pkCSM webserver. Table S4: prediction of toxicity of flavonoids inhibiting metabolic enzymes using ProTox-II. Table S5: ADMET molecular descriptors of selected flavonoids designed to inhibit SARS-CoV-2 by swissADME webserver. Table S6: GOLD fitness score, binding energy, and protein-ligand interaction of natural metabolites with spike protein RBD region. Table S7: measurements of absorbance at 450 nm and calculation of % of hACE2 bound to the S1-RBD, detected by an anti-Human HRP antibody and TMB (*n* = 3). Figure S1: 2D and 3D structures of malvidin, tomentin E, and neobavaisoflavone complexed with SARS-CoV-2 spike protein. Figure S2: binding curve of hACE2 receptor to the S1-RBD protein of SARS-CoV-2 in the presence of a wide concentration range of crude extracts from red grape as determined by ELISA.
