# Supplementary material for: Molecular Docking and Dynamics Simulation of Several Flavonoids Predict Cyanidin as an Effective Drug Candidate against SARS-CoV-2 Spike Protein
Source: Adv Pharmacol Pharm Sci. 2022 Nov 9;2022:3742318. doi: 10.1155/2022/3742318 (PMC9668477; doi:10.1155/2022/3742318)
Supplement: Supplementary Materials — Table S1: plant secondary metabolites (flavonoids) with their antiviral activity. Table S2: target protein with PDB ID, resolution, and description of the protein selected for docking with complexed inhibitor. Table S3: ADMET properties of selected flavonoids using pkCSM webserver. Table S4: prediction of toxicity of flavonoids inhibiting metabolic enzymes using ProTox-II. Table S5: ADMET molecular descriptors of selected flavonoids designed to inhibit SARS-CoV-2 by swissADME webserver. Table S6: GOLD fitness score, binding energy, and protein-ligand interaction of natural metabolites with spike protein RBD region. Table S7: measurements of absorbance at 450 nm and calculation of % of hACE2 bound to the S1-RBD, detected by an anti-Human HRP antibody and TMB (n = 3). Figure S1: 2D and 3D structures of malvidin, tomentin E, and neobavaisoflavone complexed with SARS-CoV-2 spike protein. Figure S2: binding curve of hACE2 receptor to the S1-RBD protein of SARS-CoV-2 in the presence of a wide concentration range of crude extracts from red grape as determined by ELISA. [file 3742318.f1.docx]

**Molecular Docking and Dynamics Simulation of Several Flavonoids Predict Cyanidin as an Effective Drug Candidate Against SARS-CoV-2 Spike protein**

Asmita Shrestha^1^, Rishab Marahatha^1,2^, Saroj Basnet^3^, Bishnu P. Regmi^4^, Saurav Katuwal^1^, Salik Ram Dahal^2^, Khaga Raj Sharma^1^, Achyut Adhikari^1^, Ram Chandra Basnyat^1,^ and Niranjan Parajuli^1, *^

^1^Central Department of Chemistry, Tribhuvan University, Kirtipur, Kathmandu, 44618, Nepal

^2^Department of Chemistry, Oklahoma State University, Stillwater, OK, 74078, USA

^3^Center for Drug Design and Molecular Simulation Division, Cancer Care and Research Center, Kathmandu, Nepal

^4^Department of Chemistry, Florida Agricultural and Mechanical University, Tallahassee, FL, 32307, USA

^*^Corresponding author: niranjan.parajuli@cdc.tu.edu.np

**Table S1** Plant secondary metabolites (flavonoids) with their antiviral activity

| **No.** | **Compound** | **Potential Secondary metabolites** | **IC50 value (in µM)** | **Target site/Virus** | **Citation** |
| --- | --- | --- | --- | --- | --- |
| 1. | Flavones | Scutellarein | 5.8 µM  0.86 µM (nsp13) | SARS-CoV | <https://doi.org/10.1016/j.bmcl.2012.04.081>  <https://doi.org/10.1016/j.bcp.2012.08.012> |
| 2. |  | Wogonin | 2.1 µM  (137.6 µM) CMC, HEK293T cells, CK8 assay, SPR assay | SARS-CoV  SARS-CoV-2 (binds to ACE-2) | <https://doi.org/10.1080/14756366.2021.1873977>  <https://doi.org/10.1002/ptr.7030> |
| 3. |  | Baicalin | 7.4 µM | SARS-CoV-2 | <https://doi.org/10.1101/2020.04.13.038687> |
| 4. |  | Luteolin | 20.2 µM | SARS-CoV-2 | <https://doi.org/10.1002/ptr.6873> <https://doi.org/10.1016/j.bmc.2010.09.035>  <https://doi.org/10.1080/14756366.2019.1690480> |
| 5. |  | Pectolinarin | 37.78 µM | SARS-CoV, SARS-CoV-2 | <https://doi.org/10.1080/14756366.2019.1690480> <https://doi.org/10.1111/1751-7915.13675> |
| 6. |  | Apigenin | 280.8 µM | SARS-CoV-2  SARS-CoV | <https://doi.org/10.1002/ptr.6873> <https://doi.org/10.1016/j.bmc.2010.09.035> <https://doi.org/10.1080/14756366.2019.1690480> |
| 7. |  | Amentoflavone | 8.3 µM | SARS-CoV, SARS-CoV-2 | <https://doi.org/10.1002/ptr.6873>  <https://doi.org/10.1111/1751-7915.13675> <https://doi.org/10.1080/14756366.2019.1690480>  <https://doi.org/10.1016/j.bmc.2010.09.035> |
| 8. |  | Rutin | 110 µM | SARS-CoV-2 | <https://doi.org/10.1016/j.jviromet.2012.03.020> <https://doi.org/10.1177/1934578X21991723> |
| 9. |  | Rhoifolin | 27.45 µM | SARS-CoV | <https://doi.org/10.1080/14756366.2019.1690480> |
| 10. | Flavonols | Fisetin | 85 µM | SARS-CoV-2 | <https://doi.org/10.1016/j.ejphar.2020.173759> |
| 11. |  | Galangin | 44.40 - 173.92 µM | Herpes | <https://doi.org/10.26434/chemrxiv.12115359.v1>  <https://doi.org/10.1016/S0378-8741(97)01514-6> |
| 12. |  | Herbacetin | 40.50 µM (MERS-CoV) 33.17 µM (SARS-CoV-2) | MERS-CoV, SARS-CoV, SARS-CoV-2 | <https://doi.org/10.1080/14756366.2019.1690480> <https://doi.org/10.1111/1751-7915.13675> |
| 13. |  | Myricetin | 2.7 µM | SARS-CoV | <https://doi.org/10.1016/j.bmcl.2012.04.081> |
| 14. |  | Morin | 9.42 µg/mL | MERS-CoV, SARS-CoV, Dengue Virus | <https://doi.org/10.1080/07391102.2021.1871863> <http://dx.doi.org/10.31838/srp.2020.9.118> |
| 15. |  | Tomentin A | 6.2 ± 0.04 µM(FRET) | SARS-CoV | <https://doi.org/10.1016/j.bmc.2013.03.027> |
| 16. |  | Tomentin B | 6.1 ± 0.02 µM | SARS-CoV | <https://doi.org/10.1016/j.bmc.2013.03.027> |
| 17. |  | Tomentin E | 5.0 ± 0.06 µM | SARS-CoV | <https://doi.org/10.1016/j.bmc.2013.03.027> |
| 18. | Flavanones | Naringenin | 150 µM  (FRET assay) | HCoV229, HCoVOC43, SARS-CoV-2 | <https://doi.org/10.1155/2020/5630838> |
| 19. |  | Mimulone | 14.4 µM | SARS-CoV | <https://doi.org/10.1016/j.bmc.2013.03.027> |
| 20. |  | Diplacone | 10.4 µM | SARS-CoV | <https://doi.org/10.1016/j.bmc.2013.03.027> |
| 21. |  | Hesperetin | 8.3 µM(cb) 60 µM(cf) | SARS-CoV,  SARS-CoV-2 | <https://doi.org/10.1016/j.antiviral.2005.07.002>  <https://doi.org/10.1002/ptr.6873> <https://doi.org/10.1007/s11101-020-09720-6> <https://doi.org/10.1111/cbdd.13604> |
| 22. |  | 3’-o-methyldiplacol | 9.5 ± 0.10 µM | SARS-CoV | <https://doi.org/10.1016/j.bmc.2013.03.027> |
| 23. |  | Bavachinin | 38.4 µM | SARS-CoV | <https://doi.org/10.1007/s11101-020-09720-6>  <https://doi.org/10.3109/14756366.2012.753591> |
| 24. |  | 4’-o- methyldiplacol | 9.2 µM  Cell-free assay (Fluorescence-based deubiquitination) | SARS-CoV | <https://doi.org/10.1016/j.bmc.2013.03.027> |
| 25. | Isoflavone | Daidzein | 105 µM  26.8 µM(cf);  56 µM | SARS-CoV,  SARS-CoV-2 | <https://doi.org/10.1016/j.antiviral.2005.07.002> <https://doi.org/10.1007/s11101-020-09720-6> |
| 26. |  | Corylifol A | 32.3µM | SARS-CoV | <https://doi.org/10.1007/s11101-020-09720-6> |
| 27. |  | Puerarin | 381µM | SARS-CoV | <https://doi.org/10.1007/s11101-020-09720-6> |
| 28. |  | Neobavaisoflavone | 18.3µM | SARS-CoV | <https://doi.org/10.1007/s11101-020-09720-6> |
| 29. | Flavan-3-ol | Epigallocatechin gallate | 73 µM  2.47 µg/mL | SARS-CoV  SARS-CoV-2 | <https://doi.org/10.1155/2020/5630838> <https://doi.org/10.1099/jgv.0.001574> <https://doi.org/10.1007/s10529-011-0845-8> |
| 30. | Flavanonol | Silymarin | 15.2 ± 3.53 μg/mL | MERS-CoV, SARS-CoV-2, Enterovirus 71 | <https://doi.org/10.1002/ptr.7084> <https://doi.org/10.3390/v12020184> |
| 31. | Flavans | Broussoflavan A | 92.4µM(CLpro)  20.4µM(PLpro) | SARS-CoV, MERS-CoV | <https://doi.org/10.1007/s11101-020-09720-6>  <https://doi.org/10.1080/14756366.2016.1265519> |
| 32. |  | Kazinol A | 92.4 µM(3CLpro)  66.2 µM (PLpro) | SARS-CoV | <https://doi.org/10.1007/s11101-020-09720-6>  <https://doi.org/10.1080/14756366.2016.1265519> |
| 33. |  | Kazinol B | 233.3µM(3CLpro)  31.4 µM (PLpro) | SARS-CoV | <https://doi.org/10.1007/s11101-020-09720-6>  <https://doi.org/10.1080/14756366.2016.1265519> |
| 34. | Anthocyanins | Cyanidin | 65.1 ± 14.6  µM | SARS-CoV  SARS-CoV-2 | <https://doi.org/10.1016/j.compbiolchem.2020.107408> <https://doi.org/10.1016/j.ejpe.2021.01.001> |
| 35. |  | Malvidin | 0.04573 µM (SARS-CoV-2) | SARS-CoV  SARS-CoV-2 | <https://doi.org/10.1016/j.csbj.2021.09.022> <https://doi.org/10.1016/j.ejpe.2021.01.001> |
| 36. |  | Delphinidin | 8.5 µM(DPPH) | SARS-CoV  SARS-CoV-2 | <https://doi.org/10.1016/j.ejpe.2021.01.001> |

**Table S2** Target protein with PDB ID, resolution, and description of the proteins selected for docking with complexed inhibitor

| **S.N.** | **PDB ID** | **Resolution (Å)** | **Description** | **Citation** |
| --- | --- | --- | --- | --- |
| 1. | 7NX8 | 1.95 | Crystal structure of the K417T mutant receptor binding domain of SARS-CoV-2 Spike glycoprotein in complex with COVOX-222 and EY6A Fabs | <http://doi.org/10.2210/pdb7NX8/pdb> |

**Table S3** ADMET properties of selected flavonoids by pkCSM webserver

|  | **Parameters** | **1** | **2** | **3** | **4** | **5** | **6** | **7** | **8** | **9** | **10** | **11** | **12** |
| --- | --- | --- | --- | --- | --- | --- | --- | --- | --- | --- | --- | --- | --- |
| **Absorption** | Water solubility (log mol/L) | -3.168 | -3.482 | -2.896 | -3.129 | -3.012 | -2.983 | -2.893 | -2.916 | -3.149 | -3.066 | -3.216 | -3.022 |
|  | Caco2 permeability (log Papp 10-6 cm/s) | 0.213 | 0.92 | -0.651 | 0.686 | -0.115 | 0.886 | 0.166 | -1.101 | -0.167 | 0.125 | 0.776 | 0.217 |
|  | Intestinal absorption (% absorbed) | 76.061 | 94.964 | 31.129 | 84.776 | 34.309 | 91.637 | 87.367 | 38.049 | 30.44 | 84.659 | 94.681 | 72.224 |
|  | Skin permeability (log Kp) | -2.735 | -2.762 | -2.735 | -2.735 | -2.735 | -2.746 | -2.735 | -2.735 | -2.735 | -2.735 | -2.736 | -2.735 |
| **Distribution** | VDss (Human, log L/Kg) | -0.376 | -0.216 | -0.736 | -0.04 | -0.209 | -0.176 | -1.009 | -0.019 | 0.03 | 0.018 | -0.172 | 0.075 |
|  | BBB Permeability (logBB) | -1.386 | 0.041 | -1.91 | -1.291 | -2.513 | -1.048 | -2.206 | -2.88 | -2.327 | -1.23 | -1.115 | -1.652 |
|  | CNS Permeability (log PS) | -2.598 | -2.298 | -4.637 | -2.43 | -5.48 | -2.247 | -3.477 | -5.98 | -5.327 | -2.394 | -2.257 | -3.542 |
| **Metabolism** | CYP1A2 | Yes | Yes | No | Yes | No | Yes | No | No | No | Yes | Yes | Yes |
|  | CYP2C19 | No | Yes | No | No | No | Yes | No | No | No | No | Yes | No |
|  | CYP2C9 | Yes | Yes | No | Yes | No | Yes | No | No | No | Yes | Yes | No |
|  | CYP2D6 | No | No | No | No | No | No | No | No | No | No | No | No |
|  | CYP3A4 | Yes | Yes | No | Yes | No | Yes | No | No | No | Yes | Yes | No |
| **Excretion** | Renal OCT2 substrate clearance | No | No | No | No | No | No | No | No | No | No | No | No |
|  | Total Clearance (logml/min/kg) | 0.548 | 0.429 | 0.286 | 0.618 | 0.536 | 0,674 | 0.684 | 0.13 | 0.495 | 0.482 | 0.377 | 0.535 |
| **Toxicity** | Ames Toxicity | No | No | No | No | No | No | No | No | No | No | No | No |
|  | Hepatotoxicity | No | No | No | No | No | No | No | No | No | No | No | No |
|  | Rat Oral Toxicity  (LD50) | 2.505 | 2.953 | 2.452 | 2.537 | 2.805 | 2.406 | 2.474 | 2.526 | 2.897 | 2.598 | 2.402 | 2.343 |

|  | **Parameters** | **13** | **14** | **15** | **16** | **17** | **18** | **19** | **20** | **21** | **22** | **23** | **24** |
| --- | --- | --- | --- | --- | --- | --- | --- | --- | --- | --- | --- | --- | --- |
| **Absorption** | Water solubility (log mol/L) | -2.959 | -3.119 | -4.156 | -4.746 | -4.385 | -3.338 | -4.68 | -4.191 | -3.592 | -3.94 | -5.569 | -4.186 |
|  | Caco2 permeability (log Papp 10-6 cm/s) | 0.383 | 0.896 | 1.201 | 1.367 | 1.114 | 1.117 | 1.018 | 0.939 | 0.93 | 0.544 | 1.107 | 0.962 |
|  | Intestinal absorption (% absorbed) | 62.597 | 70.913 | 84.425 | 89.016 | 77.239 | 89.969 | 90.287 | 84.205 | 72.766 | 76.904 | 94.657 | 76.952 |
|  | Skin permeability (log Kp) | -2.735 | -2.735 | -2.737 | -2.755 | -2.735 | -2.813 | -2.757 | -2.736 | -2.745 | -2.735 | -2.892 | -2.735 |
| **Distribution** | VDss (Human, log L/Kg) | 0.469 | 0.299 | 0.613 | 0.517 | 0.851 | -0.083 | 0.159 | 0.139 | -0.07 | 0.651 | 0.325 | 0.664 |
|  | BBB Permeability (logBB) | -1.717 | -1.573 | -1.415 | -1.343 | -1.396 | -1.013 | -1.056 | -1.287 | -1.121 | -1.246 | -0.145 | -1.311 |
|  | CNS Permeability (log PS) | -3.726 | -3.39 | -3.13 | -3.034 | -3.269 | -2.291 | -1.995 | -2.972 | -3.099 | -3.109 | -1.858 | -3.133 |
| **Metabolism** | CYP1A2 | Yes | Yes | No | No | No | Yes | No | No | Yes | No | Yes | No |
|  | CYP2C19 | Yes | No | No | No | No | Yes | Yes | Yes | Yes | No | Yes | No |
|  | CYP2C9 | No | No | No | Yes | No | No | Yes | Yes | No | No | Yes | No |
|  | CYP2D6 | No | No | No | No | No | No | No | No | No | No | No | No |
|  | CYP3A4 | No | No | No | Yes | No | No | Yes | No | No | No | Yes | No |
| **Excretion** | Renal OCT2 substrate clearance | No | No | No | No | No | No | No | No | No | No | No | No |
|  | Total Clearance (logml/min/kg) | 0.612 | 0.681 | 0.117 | 0.205 | 0.163 | 0.08 | 0.683 | 0.389 | 0.141 | 0.422 | 0.148 | 0.467 |
| **Toxicity** | Ames Toxicity | No | No | No | No | Yes | No | No | No | Yes | No | No | No |
|  | Hepatotoxicity | No | No | No | No | xx | No | No | No | No | No | No | No |
|  | Rat Oral Toxicity  (LD50) | 2.167 | 2.019 | 2.795 | 2.815 | 2.655 | 1.882 | 2.338 | 2.271 | 2.258 | 2.323 | 2.343 | 2.411 |

|  | **Parameters** | **25** | **26** | **27** | **28** | **29** | **30** | **31** | **32** | **33** | **34** | **35** | **36** |
| --- | --- | --- | --- | --- | --- | --- | --- | --- | --- | --- | --- | --- | --- |
| **Absorption** | Water solubility (log mol/L) | -3.325 | -4.92 | -3.494 | -4.242 | -2.935 | -3.008 | -4.134 | -4.898 | -5.418 | -3.155 | -3.402 | -2.888 |
|  | Caco2 permeability (log Papp 10-6 cm/s) | 1.027 | 0.542 | -0.099 | 0.712 | -0.758 | 0.456 | 1.3033 | 0.78 | 1.264 | -0.067 | 0.338 | -0.217 |
|  | Intestinal absorption (% absorbed) | 92.844 | 93.342 | 55.931 | 94.31 | 45.948 | 61.337 | 79.885 | 89.342 | 90.849 | 80.203 | 71.558 | 72.936 |
|  | Skin permeability (log Kp) | -2.812 | -2.752 | -2.735 | -2.778 | -2.735 | -2.735 | -2.736 | -2.798 | -3.042 | -2.735 | -2.736 | -2.735 |
| **Distribution** | VDss (Human, log L/Kg) | -0.199 | 0.196 | -0.176 | 0.167 | 0.071 | 0.273 | 0.494 | 0.232 | 0.745 | -0.295 | 0.206 | 0.403 |
|  | BBB Permeability (logBB) | -0.154 | -0.136 | -1.762 | -0.09 | -2.361 | -1.757 | -0.964 | -0.92 | -0.034 | -1.357 | -1.56 | -1.677 |
|  | CNS Permeability (log PS) | -1.974 | -1.707 | -4.072 | -1.807 | -4.304 | -3.711 | -2.781 | -1.815 | -1.534 | -2.372 | -3.255 | -3.311 |
| **Metabolism** | CYP1A2 | Yes | Yes | No | Yes | Yes | No | No | Yes | No | Yes | Yes | Yes |
|  | CYP2C19 | Yes | Yes | No | Yes | No | No | Yes | Yes | Yes | Yes | No | No |
|  | CYP2C9 | Yes | Yes | No | Yes | No | No | Yes | Yes | Yes | Yes | Yes | Yes |
|  | CYP2D6 | No | No | No | No | No | No | No | No | No | No | No | No |
|  | CYP3A4 | No | Yes | No | Yes | No | No | No | Yes | Yes | Yes | Yes | No |
| **Excretion** | Renal OCT2 substrate clearance | No | No | No | No | No | No | No | No | No | No | No | No |
|  | Total Clearance (logml/min/kg) | 0.205 | 0.309 | 0.144 | 0.242 | 0.459 | 0.056 | 0.378 | 0.548 | 0.384 | 0.651 | 0.81 | 0.655 |
| **Toxicity** | Ames Toxicity | Yes | No | No | No | Yes | Yes | No | No | No | Yes | No | Yes |
|  | Hepatotoxicity | No | No | Yes | No | No | No | Yes | No | No | No | No | No |
|  | Rat Oral Toxicity  (LD50) | 1.933 | 1.926 | 3.047 | 2.077 | 2.823 | 2.631 | 2.641 | 2.028 | 2.273 | 2.662 | 2.393 | 2.792 |

***Value Range : Solubility****: Optimal (higher than -4log mol/L) ,* ***Caco-2 Permeability:*** *Optimal (higher than -5.15 Log unit or -4.70 or -4.80) ,* ***Intestinal Absorption****: >30% Perfectly absorbed ,* ***VD (Volume Distribution)****: Optimal (0.04-20 L/Kg) ,* ***BBB (Blood Brain Barrier):*** *( BB ratio >=0.1: BBB+ ; BB ratio <0.1: BBB-) ,* ***Total Clearance:*** *>15 ml/min/kg: High; 5 ml/min/kg< CL< 15ml/min/kg: Moderate; <5 ml/min/kg: Low,* ***LD50( LD50 of acute toxicity):*** *High-toxicity: (1-50 mg/kg); Moderate-toxicity: (51-500 mg/kg); Low-Toxicity: (501-5000 mg/kg)*

**Table S4** Prediction of toxicity of flavonoids inhibiting metabolic enzymes using ProTox-II

| **Compound** | **LD_50_ mg/Kg** | **Toxicity class** | **Active Target** | **Probability** |
| --- | --- | --- | --- | --- |
| Scutellarein(1) | 3919 | 5 | Aryl hydrocarbon Receptor (AhR)  Estrogen Receptor Alpha (ER)  Estrogen Receptor Ligand Binding Domain (ER-LBD)  Mitochondrial Membrane Potential (MMP) | 0.91  0.87  0.95  1.0 |
| Wogonin(2) | 3919 | 5 | Aryl hydrocarbon Receptor (AhR)  Estrogen Receptor Alpha (ER)  Estrogen Receptor Ligand Binding Domain (ER-LBD)  Mitochondrial Membrane Potential (MMP) | 0.97  0.88  0.89  0.92 |
| Baicalin(3)  Luteolin(4)  Pectolinarin(5)  Apigenin(6)  Amentoflavone(7)  Rutin(8)  Rhoifolin(9)  Fistein (10)  Galangin(11)  Herbacetin(12)  Myricetin(13)  Morin(14)  Tomentin A(15)  Tomentin B(16)  Tomentin E(17)  Naringenin(18)  Mimulone(19)  Diplacone (20)  Hesperetin(21)  3’-O-Methyl Diplacol(22)  Bavachinin(23)  4’-O-Methyl Diplacol(24)  Daidzein(25)  Corylifol A(26)  Puerarin(27)  Neobavaisoflavone(28)  Epigallocatechin gallate(29)  Silymarin(30)  Broussoflavan A(31)  Kazinol A(32)  Kazinol B(33)  Cyanidin (34)  Malvidin(35)  Delphinidin(36) | 5000  3919  5000  2500  3919  5000  5000  159  3919  3919  159  3919  2000  2000  10000  2000  2000  2000  2000  2000  2000  2000  2430  2500  382  2500  1000  2000  2500  2500  500  5000  5000  5000 | 5  5  5  5  5  5  5  3  5  5  3  5  4  4  6  4  4  4  4  4  4  4  5  5  4  5  4  4  5  5  4  5  5  5 | -  Aryl hydrocarbon Receptor (AhR)  Estrogen Receptor Alpha (ER)  Estrogen Receptor Ligand Binding Domain (ER-LBD)  Mitochondrial Membrane Potential (MMP)  Immunotoxicity  Aryl hydrocarbon Receptor (AhR)  Estrogen Receptor Alpha (ER)  Estrogen Receptor Ligand Binding Domain (ER-LBD)  Peroxisome Proliferator-Activated Receptor Gamma (PPAR-Gamma)  Mitochondrial Membrane Potential (MMP)  Phosphoprotein (Tumor Suppressor) p53  ATPase family AAA domain-containing protein 5 (ATAD5)  Aryl hydrocarbon Receptor (AhR)  Mitochondrial Membrane Potential (MMP)  ATPase family AAA domain-containing protein 5 (ATAD5)  Immunotoxicity  Immunotoxicity  Carcinogenicity  Aryl hydrocarbon Receptor (AhR)  Estrogen Receptor Ligand Binding Domain (ER-LBD)  Mitochondrial Membrane Potential (MMP)  Aryl hydrocarbon Receptor (AhR)  Estrogen Receptor Alpha (ER)  Estrogen Receptor Ligand Binding Domain (ER-LBD)  Mitochondrial Membrane Potential (MMP)  Aromatase  Aryl hydrocarbon Receptor (AhR)  Estrogen Receptor Alpha (ER)  Estrogen Receptor Ligand Binding Domain (ER-LBD)  Mitochondrial Membrane Potential (MMP)  Aryl hydrocarbon Receptor (AhR)  Estrogen Receptor Alpha (ER)  Estrogen Receptor Ligand Binding Domain (ER-LBD)  Mitochondrial Membrane Potential (MMP)  Aryl hydrocarbon Receptor (AhR)  Aromatase  Estrogen Receptor Alpha (ER)  Estrogen Receptor Ligand Binding Domain (ER-LBD)  Mitochondrial Membrane Potential (MMP)  Immunotoxicity  Immunotoxicity  Immunotoxicity  Estrogen Receptor Alpha (ER)  Mitochondrial Membrane Potential (MMP)  -  Immunotoxicity  Immunotoxicity  Immunotoxicity  Immunotoxicity  Immunotoxicity  Aryl hydrocarbon Receptor (AhR)  Aromatase  Estrogen Receptor Alpha (ER)  Estrogen Receptor Ligand Binding Domain (ER-LBD)  Mitochondrial Membrane Potential (MMP)  ATPase family AAA domain-containing protein 5 (ATAD5)  Immunotoxicity  Mitochondrial Membrane Potential (MMP)  -  Immunotoxicity  Mitochondrial Membrane Potential (MMP)  -  Immunotoxicity  Aryl hydrocarbon Receptor (AhR) | -  0.91  0.87  0.95  1.0  0.99  1.0  1.0  1.0  1.0  1.0  1.0  0.96  0.83  0.78  0.77  0.98  0.91  0.71  0.84  0.86  0.82  1.0  1.0  0.95  1.0  0.96  0.91  0.87  0.95  1.0  0.91  0.87  0.95  1.0  1.0  0.96  1.0  0.95  1.0  0.99  0.99  0.99  0.74  0.74  -  0.76  0.90  0.94  0.91  0.94  1.0  0.71  1.0  1.0  0.91  1.0  0.95  0.70  -  0.96  0.76  -  0.97  0.99  0.94  0.87  0.99  0.79  0.92  0.82  0.83  0.79  0.92 |

**Table S5** ADME molecular descriptors of selected flavonoids designed to inhibit SARS-CoV-2 by Swiss ADME webserver

| **S.N.** | **Compound** | **MW (g/mol)** | **Log p** | **HBA** | **HBD** | **TPSA ( Å² )** | **RB** | **Molar refractivity** | **No. of heavy atoms** |
| --- | --- | --- | --- | --- | --- | --- | --- | --- | --- |
| 1. | Scutellarein | 286.24 | 2.08 | 6 | 4 | 111.13 | 1 | 76.01 | 21 |
| 2. | Wogonin | 284.26 | 2.55 | 5 | 2 | 79.90 | 2 | 78.46 | 21 |
| 3. | Baicalin | 446.36 | 1.58 | 11 | 6 | 187.12 | 4 | 106.72 | 32 |
| 4 | Luteolin | 286.24 | 1.86 | 6 | 4 | 111.13 | 1 | 76.01 | 21 |
| 5. | Pectolinarin | 622.57 | 3.41 | 15 | 7 | 227.20 | 8 | 148.29 | 44 |
| 6. | Apigenin | 270.24 | 1.89 | 5 | 3 | 90.90 | 1 | 73.99 | 20 |
| 7. | Amentoflavone | 538.46 | 3.06 | 10 | 6 | 181.80 | 3 | 146.97 | 40 |
| 8. | Rutin | 610.52 | 0.46 | 16 | 10 | 269.43 | 6 | 141.38 | 43 |
| 9. | Rhoifolin | 578.52 | -0.64 | 14 | 8 | 228.97 | 6 | 137.33 | 41 |
| 10. | Fisetin | 286.24 | 1.55 | 6 | 4 | 111.13 | 1 | 76.01 | 21 |
| 11. | Galangin | 270.24 | 2.08 | 5 | 3 | 90.90 | 1 | 73.99 | 20 |
| 12. | Herbacetin | 302.24 | 1.50 | 7 | 5 | 131.36 | 1 | 78.03 | 22 |
| 13. | Myricetin | 318.24 | 0.79 | 8 | 6 | 151.59 | 1 | 80.06 | 23 |
| 14. | Morin | 302.24 | 1.20 | 7 | 5 | 131.36 | 1 | 78.03 | 22 |
| 15. | Tomentin A | 442.50 | 3.59 | 7 | 4 | 116.45 | 5 | 120.61 | 32 |
| 16. | Tomentin B | 456.53 | 3.98 | 7 | 3 | 105.45 | 6 | 125.08 | 33 |
| 17. | Tomentin E | 472.53 | 3.24 | 8 | 4 | 125.68 | 6 | 126.24 | 34 |
| 18. | Naringenin | 272.25 | 1.84 | 5 | 3 | 86.99 | 1 | 71.57 | 20 |
| 19. | Mimulone | 408.49 | 4.80 | 5 | 3 | 86.99 | 6 | 118.85 | 30 |
| 20. | Diplacone | 424.49 | 4.42 | 6 | 4 | 107.22 | 6 | 120.87 | 31 |
| 21. | Hesperetin | 302.28 | 1.91 | 6 | 3 | 96.22 | 2 | 78.06 | 22 |
| 22. | 3’-o-methyldiplacol | 454.51 | 4.07 | 7 | 4 | 116.45 | 7 | 126.51 | 33 |
| 23. | Bavachinin | 338.40 | 3.92 | 4 | 1 | 55.76 | 4 | 97.74 | 25 |
| 24. | 4’-o-methyldiplacol | 454.51 | 4.32 | 7 | 4 | 116.45 | 7 | 126.51 | 33 |
| 25. | Daidzein | 254.24 | 1.77 | 4 | 2 | 70.67 | 1 | 71.97 | 19 |
| 26. | Corylifol A | 390.47 | 5.15 | 4 | 2 | 70.67 | 6 | 119.25 | 29 |
| 27. | Puerarin | 416.38 | 0.23 | 9 | 6 | 160.82 | 3 | 104.59 | 30 |
| 28. | Neobavaisoflavone | 322.35 | 3.74 | 4 | 2 | 70.67 | 3 | 95.69 | 24 |
| 29. | Epigallocatechin gallate | 458.37 | 1.53 | 11 | 8 | 197.37 | 4 | 112.06 | 33 |
| 30. | Silymarin | 482.44 | 1.59 | 10 | 5 | 155.14 | 4 | 120.55 | 57 |
| 31. | Broussoflavan A | 426.50 | 3.42 | 6 | 4 | 99.38 | 3 | 118.97 | 31 |
| 32. | Kazinol A | 394.50 | 5.09 | 4 | 3 | 69.92 | 5 | 118.59 | 59 |
| 33. | Kazinol B | 392.49 | 4.93 | 4 | 2 | 58.92 | 3 | 116.97 | 57 |
| 34. | Cyanidin | 287.24 | 0.56 | 6 | 5 | 114.29 | 1 | 76.17 | 32 |
| 35. | Malvidin | 331.30 | 0.71 | 7 | 4 | 112.52 | 3 | 87.13 | 39 |
| 36. | Delphinidin | 303.24 | 0.13 | 7 | 6 | 134.52 | 1 | 78.20 | 33 |

**Table S6** GOLD fitness score, binding free energy, and protein-ligand interaction of natural metabolites with spike protein RBD region (7NX8)

| Compounds | Gold Fitness Score | Interacting residues | Bond length (Å) |
| --- | --- | --- | --- |
| Scutellarein | 42.01 | Glu 340  Arg 346  Ser 399 | 2.15  2.20  2.08 |
| Wogonin | 36.30 | Asn 343 | 2.02 |
| Luteolin | 46.27 | Glu 340  Thr 345  Arg 355 | 2.24  2.19  1.97 |
| Apigenin | 48.66 | Thr 345  Arg 355 | 1.87  1.96 |
| Fisetin | 46.33 | Glu 340  Val 341  Ser 399 | 4.24  4.55  2.30 |
| Galangin | 48.35 | Asp 467  Tyr 473 | 1.79  4.38 |
| Herbacetin | 47.02 | Ser 371  Trp 436  Asn 437 | 2.08  2.06  2.08 |
| Morin | 42.86 | Glu340  Glu 340  Ser 399 | 2.37  3.15  2.22 |
| Tomentin A | 41.32 | Phe 342  Thr 345 | 2.45  4.27 |
| Tometin B | 52.18 | Gly 339  Thr 345  Arg 355 | 2.15  2.41  2.12 |
| Naringenin | 42.82 | Val 341  Thr 345  Ser 399 | 4.53  2.04  2.10 |
| Diplacone | 45.49 | Asn 343  Arg 509 | 1.97  2.49 |
| Hesperetin | 42.78 | Ser 371  Ser 373 | 2.13  2.31 |
| 3’-o-Methyldiplacol | 45.26 | Ser 373 | 2.13 |
| Bavachinin | 42.58 | Ser 373  Asn 437 | 3.75  3.94 |
| Daidzein | 45.34 | Asp 467  Ser 469  Gln 474 | 1.96  3.67  2.40 |
| Puerarin | 48.12 | Ser 371  Asn 437  Asn 440  Leu 441  Leu 441  Arg 509 | 2.44  2.19  2.20  3.93  3.94  2.29 |
| Silymarin | 32.21 | Phe 342  Asn 439  Asn 440 | 1.88  2.31  2.43 |
| Broussoflavan | 47.03 | Asn 370  Ser 371  Ser 373 | 1.97  2.20  4.86 |
| Kazinol B | 38.92 | Ala 372  Ser 373 | 3.79  4.52 |

**Table S7** Measurements of absorbance at 450 nm and calculation of % of hACE2 bound to the S1-RBD, detected by an anti-Human HRP antibody and TMB. (n=3)

| S.N. | Concentration (mg/mL) | Absorbance (mean) | % of hACE2 bound to the S1-RBD (mean) |
| --- | --- | --- | --- |
| 1. | 0.0000000 | 0.614 | 100.00000 |
| 2. | 0.0781250 | 0.423 | 68.89251 |
| 3. | 0.1562500 | 0.361 | 58.79479 |
| 4. | 0.3125000 | 0.356 | 57.98046 |
| 5. | 0.6250000 | 0.321 | 52.28013 |
| 6. | 1.2500000 | 0.316 | 51.4658 |
| 7. | 2.5000000 | 0.282 | 45.9283 |
| 8. | 5.0000000 | 0.269 | 43.8111 |
| 9. | 10.000000 | 0.223 | 36.3192 |

| **(a)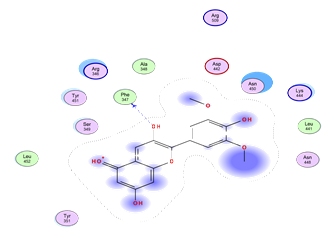** | **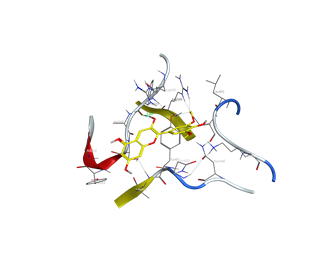** |
| --- | --- |
| **(b)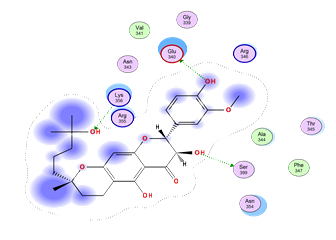** | **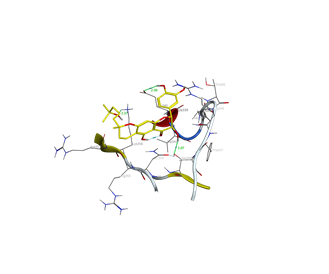** |
| **(c)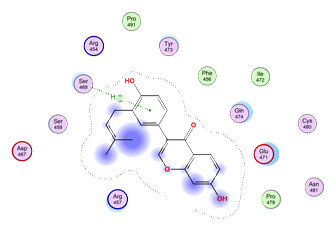** | **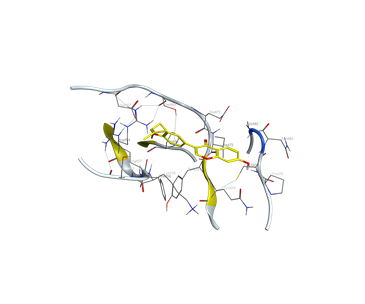** |

**Figure S1** 2D and 3D structures of Malvidin, Tomentin E, and Neobavaisoflavone complexed with SARS-CoV-2 spike protein

**Figure S2** Binding curve of hACE2 receptor to the S1-RBD protein of SARS-CoV-2 in the presence of a wide concentration range of crude extracts from red grape as determined by ELISA. The data represents mean ± SEM from n=3 samples.
